# Oritavancin a Therapeutic Option for Periprosthetic Joint Infections in Selected Cases: A Comprehensive Review

**DOI:** 10.3390/ph18081217

**Published:** 2025-08-18

**Authors:** Rares-Mircea Birlutiu, Victoria Birlutiu

**Affiliations:** 1 Department 14-Orthopedics, Anaesthesia Intensive Care Unit, Faculty of Medicine, “Carol Davila” University of Medicine and Pharmacy, 020021 Bucharest, Romania; 2 Foisor Clinical Hospital of Orthopedics, Traumatology, and Osteoarticular TB, 030167 Bucharest, Romania; 3 Faculty of Medicine, Lucian Blaga University of Sibiu, 550169 Sibiu, Romania; 4 County Clinical Emergency Hospital, 550245 Sibiu, Romania

**Keywords:** oritavancin, periprosthetic joint infection, PJI, biofilm, MRSA, lipoglycopeptide, antimicrobial therapy, comprehensive review

## Abstract

**Background:** Periprosthetic joint infections (PJIs) remain among the most challenging complications in orthopedic surgery, often requiring prolonged antibiotic therapy and complex surgical interventions. Oritavancin, a long-acting semisynthetic lipoglycopeptide approved for acute bacterial skin and skin structure infections, has emerged as a potential off-label agent in PJI treatment due to its favorable pharmacokinetic properties, potent Gram-positive coverage, and documented antibiofilm activity. **Objectives:** This comprehensive review aims to assess the current clinical and preclinical data regarding the potential use of oritavancin in the management of PJIs. Methods: A comprehensive literature search was conducted in three major databases. **Results:** Six studies were included. In vitro data demonstrated strong activity of oritavancin against methicillin-resistant *Staphylococcus aureus* and *S. epidermidis* biofilms, particularly in synergy with rifampin. Clinical reports described successful outcomes in both acute and chronic PJI cases, including those with limited surgical options. Weekly or monthly dosing regimens were well-tolerated and effective in suppressive and curative contexts. Adverse events were infrequent but included infusion-related reactions. **Conclusions:** Oritavancin represents a promising adjunct or alternative to conventional antimicrobial regimens in PJIs, particularly for outpatient management or in patients with multidrug-resistant Gram-positive infections. Further prospective studies are needed to define its role, optimal dosing, and long-term efficacy in this complex clinical setting.

## 1. Introduction

The discovery of the natural lipoglycopeptide teicoplanin after 30 years from the discovery of vancomycin in 1952, created an immense interest in the research and development of semisynthetic lipoglycopeptide antibiotics. Up to this moment, three members of this new class of antibiotics have been approved for clinical use: telavancin, dalbavancin, and oritavancin [1]. Oritavancin, as previously mentioned, is a semisynthetic lipoglycopeptide antibiotic derived from the vancomycin class of antibiotics; it has been developed to target serious Gram-positive bacterial infections. It was approved for the treatment of acute bacterial skin and skin structure infections (ABSSSI) in adult population, but its potent activity and pharmacologic profile have prompted interest in broader applications. Oritavancin exerts bactericidal effects through multiple mechanisms of action. It inhibits peptidoglycan synthesis by binding to the D-alanyl-D-alanine terminus of cell wall precursors, blocking the transglycosylation and transpeptidation steps of cell wall formation. In addition, oritavancin’s hydrophobic 4′-chlorobiphenylmethyl side chain enables it to disrupt the integrity of the bacterial cell membrane, causing depolarization and increased permeability [2]. Through these three mechanisms, oritavancin achieves rapid, concentration-dependent killing of Gram-positive organisms, including those in stationary and biofilm growth states [2].

Prosthetic joint infections (PJIs) are one of the most serious complications after total joint arthroplasty, leading to multiple revision surgeries, prolonged antibiotic therapy, and high patient morbidity and also increased healthcare costs [3,4,5]. Although the incidence rate of PJI is relatively low (around 1–2% after primary replacements), the absolute number of PJIs is increasing with the growing number of arthroplasty procedures performed in each year [3,4,5]. Large registries and recent cohort studies indicate PJI rates of ~0.5–2.3% after total hip arthroplasty (THA) [6] and around 1% after total knee arthroplasty (TKA), while shoulder arthroplasty PJIs occur in ~1–4% of cases and elbow arthroplasty infections in 1.5–12.5% [7]. Despite modern preventive measures, recurrence or re-infection rates remain significant, even after revision surgery: PJI recurs in approximately 5–15% of cases on average, with higher re-infection rates observed in knees than in hips [8]. Staphylococci (especially *Staphylococcus aureus* and *S. epidermidis*) are the predominant PJI pathogens, accounting for roughly 65–85% of cases, while Gram-negative bacilli cause 6–23% of cases [9,10,11]. Notably, multidrug-resistant organisms are an increasing concern—for example, nearly half of *S. aureus* isolates in some cohorts are methicillin-resistant [11]. Effective treatment of PJI requires a combined surgical and antimicrobial approach to eradicate infection while restoring joint function. Treatment failure rates of current strategies are likewise substantial. Two-stage exchange (the gold-standard for chronic PJI) achieves infection eradication in roughly 80–90% of patients [7], implying a 10–20% failure rate, and one-stage revision can yield similarly high success in appropriately selected cases [8]. By contrast, less invasive debridement and implant retention approaches often have markedly lower cure rates (approximately 50–70% infection control in chronic PJI) [12], underscoring the difficulty of eradicating established infections.

Systemic antibiotic therapy is as critical as surgery in PJI management and should be initiated promptly after appropriate cultures are obtained [11,13,14,15]. Empiric antibiotic regimens are chosen to broadly cover the most probable pathogens (guided by the local epidemiology of PJI) until specific culture results are available [11,13,14,15]. Periprosthetic joint infections caused by multidrug-resistant (MDR) organisms have emerged as a critical challenge in orthopedic surgery, significantly complicating management and contributing to treatment failure, recurrence, and increased mortality. Methicillin-resistant *Staphylococcus aureus* (MRSA) and methicillin-resistant coagulase-negative staphylococci (MRSE) are representing an increasing proportion [16]. MRSA alone is implicated in 10–20% of PJIs in many regions, rising to over 30% in high-endemicity areas such as Southern Europe and parts of Asia [15]. Similarly, methicillin resistance among *S. epidermidis* isolates has grown substantially, reflecting broader antimicrobial resistance trends [17].

Enterococcal PJIs, though less common (~2–8%), are increasingly caused by vancomycin-resistant strains (VRE), which present limited therapeutic options and are associated with poorer outcomes [18]. Gram-negative bacilli contribute 10–25% of PJIs globally, with MDR strains, particularly extended-spectrum β-lactamase (ESBL)-producing *Enterobacterales* and carbapenem-resistant *Pseudomonas* spp. or *Acinetobacter* spp., becoming more prevalent, especially in Asia, Africa, and Latin America [11]. A multicenter European study reported >50% of Gram-negative PJI isolates as MDR, including 13.5% carbapenem-resistant strains [11].

The impact of MDR organisms on clinical outcomes is substantial. These infections are associated with higher rates of treatment failure, reinfection, longer hospital stays, and greater need for complex surgical interventions such as two-stage exchange arthroplasty [14]. Debridement and implant retention (DAIR) strategies show significantly lower success in MRSA or VRE PJIs compared to susceptible strains [19,20], and recurrence remains high due to biofilm persistence and limited antibiotic penetration [19,20].

Current standard therapies, including vancomycin, linezolid, and daptomycin, are often constrained by suboptimal bone penetration, toxicity, resistance emergence, or limited efficacy against biofilms. These limitations underscore the urgent need for new agents. Its extended half-life allows for intermittent dosing, potentially improving adherence and reducing healthcare burdens. As MDR PJIs continue to rise globally, the exploration and clinical validation of novel agents like oritavancin is imperative. Ongoing research is targeting the PJI challenges of biofilm and resistance, ranging from improved diagnostic techniques to novel antibiofilm including coatings for implants, with the goal of improving cure rates and preserving joint function in this difficult clinical problem. In this context, this comprehensive review aims to evaluate the current evidence regarding the pharmacological characteristics, clinical efficacy, and potential therapeutic role of oritavancin in the management of prosthetic joint infections. A long-acting lipoglycopeptide with potent in vitro activity against MRSA, VRE, and biofilm-associated staphylococci, Oritavancin has shown promise in early reports for salvage and suppressive PJI therapy.

## 2. Results and Discissions

The database search yielded a total of 31 records. After removal of duplicates, 16 unique records were screened by title and abstract. Of these, four were excluded as irrelevant. Nine full-text articles were assessed for eligibility, and four were excluded: four were not related to periprosthetic joint infections. One study was identified through other sources and included in this comprehensive review. Ultimately, six studies were included in the qualitative synthesis of this comprehensive review.

### 2.1. Pharmacokinetics

Pharmacokinetic studies revealed a distinct characteristic of oritavancin, mainly its long plasma half-life and the possibility of extending the period between dosing. The terminal elimination half-life of oritavancin is approximately 245 h (about 10 days), with a prolonged tail phase that in some studies extends up to ~393 h [21]. This increased half-life allows oritavancin to be administered as a single-dose regimen for ABSSSI. The standard FDA-approved dosing is 1200 mg given as a one-time intravenous infusion (typically administered over 3 h) [21]. A newer formulation has been developed to allow the same 1200 mg dose to be infused over 1 h (brand name Kimyrsa [22]). The new formulation is characterized by the reduction of the intravenous infusion volume (from 1000 mL to 250 mL), shorter infusion time (from 3 h to 1 h), and also providing pharmacies/doctors with increased flexibility in preparation of the infusion (from 5% dextrose in sterile water to either normal saline or 5% dextrose in sterile water) compared with the initial formulation [22]. Oritavancin’s extended half-life and concentration-dependent killing support increased dosing intervals; for off-label treatment of different types of infections, regimens involving weekly dosing have been employed (for example, 1200 mg initial dose followed by 800 mg once weekly for several weeks) [23]. Pharmacokinetic studies revealed that oritavancin is a highly protein-bound antibiotic; approximately 85% binds to proteins. It has also been highlighted that it exhibits a large volume of distribution (~80–87 L) into different tissues, from penetrating effectively into skin and soft tissues as expected for ABSSSI, into achieving good concentrations in bone, synovial fluid, and macrophages within reticuloendothelial system organs [21,22,23,24]. Animal models demonstrate that oritavancin is rapidly distributed into bone tissue, achieving bone concentrations that exceed simultaneous plasma levels (bone/plasma ratios 110–310%) and remain above the MIC_90_ for *S. aureus* for at least 7 days post-dose [25]. This extensive tissue distribution is a major advantage for the management of different types of infections like bone and joint infections including periprosthetic joint infections. Oritavancin is not significantly metabolized by, nor reliant upon, the liver’s cytochrome P450 system. Its primary clearance occurs through slow plasma elimination and tissue uptake rather than renal excretion. No dosage adjustments are required for patients with mild to moderate renal or hepatic impairment [21].

From a mechanism of action, oritavancin has a multimodal one that seems to be well-suited to PJIs. It binds to the D-alanyl-D-alanine terminus of peptidoglycan precursors, blocking both the transglycosylation (polymerization) and transpeptidation (cross-linking) steps of Gram-positive cell wall synthesis. In addition, its hydrophobic N-alkyl side chain enables oritavancin to disrupt bacterial membrane integrity by depolarizing the membrane and increasing permeability. Oritavancin’s multi-target action also extends to inhibition of RNA synthesis, distinguishing it from vancomycin [25].

One pharmacologic consideration is that oritavancin can cause temporary interference with laboratory tests. It may artificially prolong certain coagulation assays (activated partial thromboplastin time, prothrombin time) for up to 120 h after dosing, due to its binding to assay reagents. In this context clinicians should be aware of this interaction, although it is a laboratory artifact and not due to actual coagulopathy [21].

Overall, the pharmacokinetic profile of oritavancin, marked by an extremely long half-life, high tissue penetration, and convenient dosing, makes it well-suited for outpatient therapy and improved compliance in prolonged treatment courses as in the case of periprosthetic joint infections.

### 2.2. In Vitro Activity of Oritavancin Against Biofilms

Yan et al. assessed the in vitro activity of oritavancin against biofilms formed by 185 staphylococcal isolates obtained from PJI cases comprising methicillin-resistant and methicillin-susceptible strains of *S. aureus* and *S. epidermidis*. These isolates were derived from patients treated at Mayo Clinic between 1996 and 2016, with infections predominantly involving knee and hip endoprostheses. Oritavancin demonstrated potent activity against planktonic staphylococci, with MIC50 values of 0.03 μg/mL for MRSA, MSSA, and MSSE, and 0.06 μg/mL for MRSE. MIC90 was uniformly 0.12 μg/mL across all groups. Notably, eight *S. epidermidis* isolates (six MRSE) exhibited elevated MIC values above 0.12 μg/mL. All tested isolates demonstrated biofilm-forming capability. Oritavancin showed effective inhibition of staphylococcal biofilms, with MBIC50 values of 1 μg/mL for all staphylococcal subgroups tested, while MBIC90 values were 2 μg/mL for *S. aureus* and 4 μg/mL for *S. epidermidis*. The MBBC50 values were consistently 2 μg/mL, whereas MBBC90 values varied, being 4 μg/mL for MRSA, MSSA, and MSSE, and higher at 8 μg/mL for MRSE. The isolates exhibiting elevated planktonic MIC values also demonstrated increased biofilm resistance. These findings indicate that oritavancin has robust in vitro activity against staphylococcal biofilms from PJIs, underscoring its potential utility in treating such biofilm-related infections [26].

Yan et al. evaluated the antibiofilm activity of oritavancin combined with rifampin, gentamicin, or linezolid against biofilms formed by ten methicillin-resistant *Staphylococcus aureus* (MRSA) isolates from PJIs. These isolates were collected from patients treated at Mayo Clinic between 2000 and 2016. The authors performed biofilm time-kill assays using Teflon coupons inoculated with bacterial isolates, treated with antimicrobials individually or in combinations at either minimum biofilm bactericidal concentrations (MBBCs) or peak achievable plasma concentrations (*fC_max_*). Bactericidal activity was defined as at least a 3-log reduction in biofilm bacterial density compared to baseline, and synergy was defined as at least a 2-log reduction compared to the most active single agent. Oritavancin combined with rifampin demonstrated statistically significant reductions in biofilm bacterial density at 24 h compared to either agent alone for all ten isolates (*p* ≤ 0.001), achieving synergy in 80% of cases. When combined with gentamicin, oritavancin exhibited bactericidal activity against all isolates but synergy in only 20%. Notably, gentamicin alone showed bactericidal activity in 80% of isolates. The combination of oritavancin and linezolid demonstrated significant reductions for 80% of isolates (*p* ≤ 0.01), though synergy was achieved in only 30%. The observed synergy between oritavancin and rifampin likely arises from complementary mechanisms: oritavancin disrupts bacterial cell walls and membranes, facilitating rifampin penetration, and may itself inhibit bacterial RNA synthesis, enhancing rifampin’s effect. The maximum concentrations of the antimicrobial assessed by the authors were 16 μg/ml for oritavancin and 4 μg/ml for rifampin. The MIC and MBBC values for the isolated strains in this article are highlighted in Table 1 [27].

### 2.3. The Use in the Field of Periprosthetic Joint Infections

One particularly promising off-label application is in prosthetic joint infections, infections requiring prolonged therapy. Oritavancin’s characteristics, such as its long half-life, bone penetration, and activity against MRSA and enterococci, are well suited to these conditions. Several studies have documented favorable outcomes using oritavancin for the management of PJIs.

Krsak and colleagues presented a retrospective, multicenter observational case series evaluating oritavancin use for therapeutic and suppressive management of bone and joint infections caused by vancomycin-resistant *Enterococcus faecium* (VRE), including osteomyelitis, native septic arthritis, myositis, and prosthetic joint infections, from December 2014 through April 2024. Among these cases, one involved a 64-year-old male patient with a PJI of a right total knee arthroplasty due to VRE (oritavancin MIC: 0.25 mg/L), who received weekly oritavancin (1200 mg, 8 doses total) following prior treatment with daptomycin, linezolid, and cefepime, alongside concurrent meropenem therapy and subsequent phage therapy. The patient experienced recurrence of knee stiffness and persistent VRE infection but no adverse drug events within a follow-up period of 195 days. Additionally, the authors reported a suppressive therapy case involving a 68-year-old female patient with a right hip PJI managed successfully with monthly infusions of oritavancin (1200 mg/month for 22 months), achieving sustained symptom-free status without adverse reactions throughout the 22-month follow-up [28].

Buonomo et al. described the case of an elderly male patient in his eighties known with high blood pressure, obesity, chronic ischemic heart disease, chronic obstructive pulmonary disease, chronic kidney disease (stage 3b according to KDIGO classification (Kidney Disease Improving Global Outcomes 2012)), and peripheral vascular disease, who developed a late-onset PJI of the right shoulder (reverse total shoulder prosthetic surgery stabilized by metal hoop for right humeral fracture for which in 2021 the patient underwent surgical intervention) due to methicillin-resistant *Staphylococcus epidermidis* (MRSE). The patient had contraindications for surgical exchange and limited oral antibiotic options suitable for long-term suppressive therapy. In this case, the prosthesis was retained, and the patient underwent treatment with outpatient-administered sequential doses of oritavancin (1200 mg per dose), guided by therapeutic drug monitoring (TDM). Over a period of 28 weeks, the patient received a total of ten doses. During his therapy, the patient achieved clinical resolution, experiencing complete relief of pain, recovery of preinfection joint mobility, and avoiding any further hospitalizations or surgical interventions. No adverse reactions were noted throughout treatment. Currently, suppressive oritavancin therapy continues, with doses of 1200 mg administered every 28 days, guided by TDM. At the time of reporting the case by the authors, after ten doses over the course of 28 weeks, the patient remains symptom-free with fully restored shoulder mobility. The authors plan to perform a PET-CT scan after 48 weeks to assess the possibility of safely discontinuing antimicrobial therapy [29].

Bandaranayake et al. reported a retrospective analysis involving 95 patients who received oritavancin for various infections, among whom 5 patients (5.2%) had PJIs. The pathogens isolated from these PJI cases included coagulase-negative staphylococci (2 cases), *Corynebacterium striatum* (1 case), *Enterococcus faecalis* along with *Corynebacterium species* (1 case), and *Cutibacterium acnes* (1 case). Among these patients, three experienced adverse reactions: a 56-year-old female developed itching, hives, and throat swelling during her first infusion, which required cessation of therapy and intravenous diphenhydramine administration; a 60-year-old female had a rash after her third dose, which appeared post-infusion and was managed with topical steroids following dermatological biopsy; and a 46-year-old female experienced shaking, chills, vomiting, and back pain during her third infusion, necessitating immediate discontinuation and administration of hydrocortisone, intravenous diphenhydramine, famotidine, and ondansetron. Although the overall study cohort demonstrated a success rate of 74%, specific outcomes for the individual PJI cases were not detailed [24].

Redell et al. analyzed data from the retrospective observational Clinical and Historic Registry and Orbactiv Medical Evaluation (CHROME) program, conducted between 2014 and 2017, involving 440 patients across 26 US sites treated with oritavancin for ABSSSI and other Gram-positive infections. Within this cohort, PJIs accounted for 9.4% (3 out of 32) of non-ABSSSI cases. Among these, two patients received a single dose of oritavancin, with one achieving clinical cure and the other experiencing treatment failure; both had extensive histories of prior antibiotic exposure including beta-lactams, vancomycin, clindamycin, and daptomycin. Additionally, one patient received multiple doses (1200 mg × 2, administered every 14 days), successfully achieving clinical cure without adverse events. The authors noted that in the two patients treated with single-dose oritavancin for septic arthritis/synovitis, treatment failed in one case due to the presence of a retained foreign body (the prosthesis) [30].

Nguyen et al. reported the case of a patient who developed an acute postoperative PJI, due to vancomycin-susceptible *Enterococcus faecalis*, occurring 21 days after an initial right hip total joint arthroplasty for severe osteoarthritis. The patient underwent extensive surgical exploration, employing a debridement, antibiotics, and implant retention (DAIR) approach. Initial antimicrobial therapy consisted of 10 days of combined daptomycin and ampicillin. The patient continued to experience pain and swelling with the inability to bear weight on the right lower extremity. Several options had been offered to the patient, including the following: DAIR, a two-stage exchange procedure, or salvage therapy with intravenous oritavancin 1200 mg weekly. The patient opted for the third option; subsequently, the prosthesis was successfully retained and salvaged through outpatient administration of six sequential doses of oritavancin (1200 mg weekly), without further intra-articular irrigation or additional surgical intervention. At ten months after completing oritavancin therapy, the patient remained symptom-free and was ambulating independently without pain or reliance on assistive devices [31].

Antony and Cooper report two patients with PJIs successfully treated using a single-stage revision procedure with an antibiotic spacer, coupled with oritavancin therapy [32].

Table 2 provides an overview of the cases published until this moment.

### 2.4. Safety

Oritavancin is approved for acute bacterial skin and skin structure infections (ABSSSI) and has been used off-label in other serious Gram-positive infections (e.g., osteomyelitis) [24]. In two phase-3 trials (Solo I and II), a single 1200-mg infusion of oritavancin was non-inferior to vancomycin and showed a similar overall incidence of adverse events [24,33]. About 50–60% of patients in these trials experienced at least one treatment-emergent adverse event (versus ~50–64% with vancomycin) [34]. The most common adverse reactions (≥3% incidence) were nausea (~10% of patients) [35], headache (~7%) [35], vomiting (~5%) [35], and diarrhea (~4%) [35]. These were generally mild to moderate. Serious adverse events were infrequent (~4–7%) and occurred at rates comparable to vancomycin [34]. The most common serious reaction reported was cellulitis (about 1.1%) in both oritavancin and vancomycin arms [36]. Mortality in trials was low and similar between groups (2 oritavancin vs. 3 vancomycin patients) [34].

#### 2.4.1. Infusion-Related and Hypersensitivity Reactions

Like other glycopeptides, oritavancin can cause infusion-related reactions. In trials, infusion site reactions (e.g., phlebitis, erythema) occurred in roughly 10% of patients, similar to vancomycin. “Red man syndrome”-like symptoms (flushing, pruritus, urticaria) have been reported during or shortly after infusion. Rare infusion reactions have included chest or back pain, chills, and tremor; these generally resolved upon slowing or stopping the infusion [35,36,37]. To minimize risk, the recommended infusion duration for oritavancin is 3 h [35,36,37]. In late 2019, the FDA updated the product label to strengthen warnings about infusion-related reactions [38]. Real-world data also indicate that ~14% of patients may experience an oritavancin-related adverse reaction, 85% of which occur during the infusion (often manageable by pausing or slowing the drip) [24]. Serious hypersensitivity reactions (including anaphylaxis) are rare but have been observed; clinicians should monitor for bronchospasm, angioedema, or rash and discontinue therapy if such reactions occur [16]. Notably, oritavancin’s long terminal half-life (~393 h) means that any severe allergic reaction could persist longer even after stopping the drug [35,36,37,38].

#### 2.4.2. Hepatotoxicity and Other Safety Considerations

Clinically significant hepatotoxicity with oritavancin appears uncommon. Transient elevations in liver enzymes were observed in trials at low rates, e.g., ALT increases in ~2–3% of patients (similar to vancomycin). Only one patient (<0.1%) on oritavancin had a marked ALT elevation >10× the upper limit of normal. No cases meeting Hy’s law criteria for severe drug-induced liver injury were identified. Oritavancin has not been associated with nephrotoxicity, and no dose adjustments are needed for renal or mild hepatic impairment [35,39].

#### 2.4.3. Drug Interactions and Laboratory Interferences

Oritavancin exhibits some important drug–laboratory and drug–drug interactions. It artificially prolongs coagulation tests by binding assay reagents: activated partial thromboplastin time (aPTT) can be prolonged for up to 120 h after a dose, and prothrombin time/INR for ~12 h. Therefore, use of IV unfractionated heparin is contraindicated for 5 days after oritavancin, and warfarin coagulation monitoring is unreliable on the day of oritavancin infusion. Patients on chronic warfarin who receive oritavancin should be observed for bleeding and have extra INR monitoring once the interference period ends. Aside from lab effects, oritavancin may affect other drugs’ metabolism. It is a nonspecific weak inhibitor of CYP2C9 and CYP2C19, and a weak inducer of CYP3A4 and CYP2D6. No significant QT prolongation or other cardiac toxicity has been attributed to oritavancin. Oritavancin’s safety profile has remained consistent in post-marketing surveillance, with no new serious safety signals prompting regulatory action as of 2025 [35,36].

When using oritavancin for the management of periprosthetic joint infection, clinicians must consider the overall clinical picture. The drug’s long half-life should definitely be taken into consideration, and means that adverse reactions, should they occur, might persist longer than in other cases. Bandaranayake et al. reported from a retrospective study conducted over a 5-year period based on 95 enrolled patients that have been followed up for 1 year, that 13% of patients on oritavancin for invasive infections experienced infusion-related reactions or other side effects requiring discontinuation [24]. Common side effects of oritavancin include headache, nausea, infusion-site reactions, and the laboratory coagulation test interference. Serious allergic reactions are uncommon, and oritavancin does not have the nephrotoxicity risk associated with vancomycin. Same authors reported the following case of infusion-related reaction in a patient with PJI. Female patient that was receiving oritavancin therapy as part of a 3-dose regimen (1200 mg initial dose followed by 800 mg × 2). Prior to infusion, the patient received prophylactic diphenhydramine 50 mg orally. During the infusion, the patient developed acute symptoms including generalized shaking, chills, nausea with vomiting, and lumbar back pain. The infusion was immediately discontinued. Emergency management included administration of hydrocortisone 100 mg IV, diphenhydramine 50 mg IV, famotidine 20 mg IV, and ondansetron 4 mg IV. The patient’s symptoms resolved following treatment [24].

### 2.5. Limitations

This comprehensive review also has several limitations. First, the overall number of published studies specifically investigating oritavancin in the context of periprosthetic joint infections remains limited, with the majority consisting of case reports, small case series, or retrospective observational studies lacking control groups, and based on off-label use. As a result, the strength of the evidence remains low. Second, substantial heterogeneity exists among the included cases in terms of pathogens involved, surgical strategies, and adjunct antimicrobial regimens, which may confound interpretation of outcomes. Third, most studies lacked standardized criteria for clinical success or failure, with outcome reporting often being descriptive and inconsistent. Finally, publication bias may be present, with successful or novel cases being more likely to be reported than failures or adverse events.

## 3. Materials and Methods

**Figure 1:**
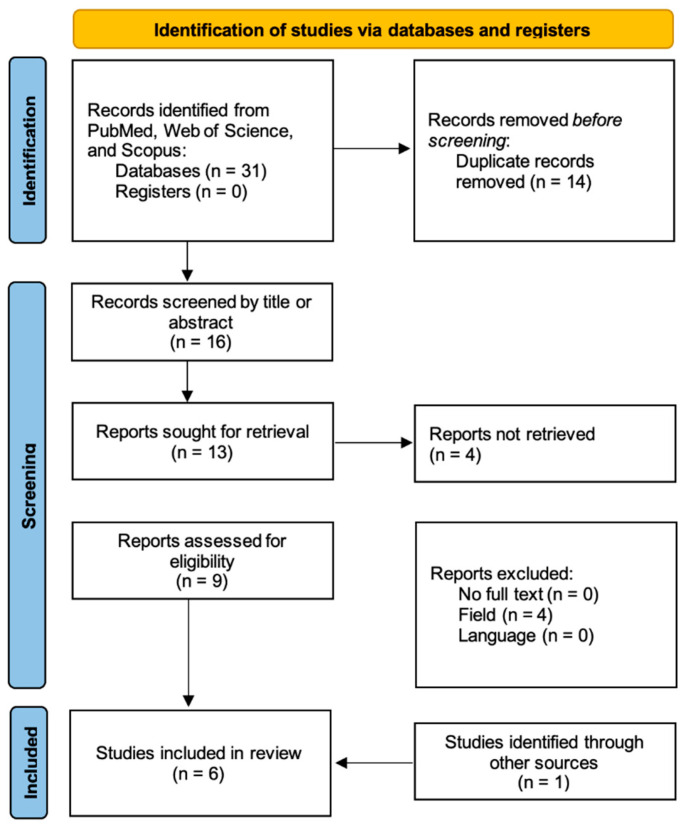
Flow diagram of included studies in the article.

For this comprehensive review, a literature search was conducted in three major databases—PubMed, Scopus, and Web of Science—to identify studies reporting the use of oritavancin in the treatment of periprosthetic joint infections, from January 1990 to June 2025. No restrictions were applied with respect to study design or therapeutic context, and studies were not limited to case reports, salvage therapy, or off-label use. Only articles published in English and involving human subjects were included. In PubMed, the following search string was used: (“oritavancin”[Mesh] OR oritavancin[tiab]) AND (“prosthetic joint infection” OR “periprosthetic joint infection” OR “periprosthetic joint infections” OR PJI OR “Prosthesis-Related Infections”[Mesh] OR prosthesis-related infection OR prosthesis related infection OR “joint prosthesis infection”)) AND (1990:3000[pdat]) AND english[la]) NOT (animals[mh] NOT humans[mh]). Equivalent queries were applied to Scopus (TITLE-ABS-KEY(oritavancin AND (“periprosthetic joint infection” OR “prosthetic joint infection” OR “prosthesis-related infection” OR “joint prosthesis infection” OR PJI))) and Web of Science (TS = (oritavancin AND (“periprosthetic joint infection” OR “prosthetic joint infection” OR “prosthesis-related infection” OR “joint prosthesis infection” OR PJI))). All identified records were imported into a reference management software and screened for duplicates. Titles and abstracts were independently reviewed by both authors to assess relevance, and full texts of potentially eligible studies were retrieved and further examined. Data extraction was performed independently by both authors. Although a formal risk of bias assessment tool was not applied due to the anticipated predominance of descriptive studies, each included article was assessed qualitatively with attention to methodological rigor, clarity of outcome reporting, and potential confounding. The study selection process is illustrated in a PRISMA flow diagram (Figure 1) [40].

## 4. Conclusions

Oritavancin has emerged as a promising off-label therapeutic option for the management of periprosthetic joint infections, providing distinct pharmacokinetic advantages, with an in vitro antibiofilm activity, and a favorable clinical outcome in select patient populations based on the limited published data until this moment. Its prolonged half-life, high protein binding, extensive tissue penetration including bone and synovial tissues, and minimal reliance on renal clearance make it particularly suitable for outpatient, long-interval dosing regimens. In vitro studies have demonstrated that oritavancin retains strong bactericidal activity against staphylococcal biofilms, including methicillin-resistant *S. aureus* and *S. epidermidis*. Combinations with rifampin or gentamicin have shown synergistic effects, enhancing its efficacy against biofilm-associated infections. Evidence from the limited real-world clinical use supports oritavancin’s role, particularly in patients with limited surgical options or contraindications to standard therapies. While its safety profile is generally favorable, infusion-related adverse events can occur and may be prolonged due to the drug’s long elimination phase.

Overall, oritavancin represents a valuable addition to the list of antimicrobial agents against difficult-to-treat PJIs based on the very limited published data and on the off-label use for the management of PJIs. While current evidence supports its potential role, definitive guidance on optimal dosing regimens, treatment duration, and combination strategies remains lacking. To ensure safe and effective integration into clinical practice, well-designed prospective clinical trials and large-scale, registry-based observational studies are essential. These efforts will help establish standardized protocols, refine patient selection, and clarify the long-term outcomes of oritavancin-based therapy in PJI management.

## Figures and Tables

**Table 1:** MIC and MBBC values for MRSA isolates published by Yan et al. [27].

Isolate of MRSA	MIC (μg/mL) of ^a^:				MBBC (μg/mL) of ^a^:			
	ORI	RIF	GEN	LZD	ORI	RIF	GEN	LZD
1	0.03	0.004	0.25	2	4	4	1	>128
2	0.03	0.008	0.25	4	4	16	0.5	>128
3	0.06	0.008	0.25	2	16	4	2	>128
4	0.06	0.008	0.25	2	4	16	0.5	>128
5	0.015	0.008	0.25	2	8	4	2	>128
6	0.12	0.008	0.25	2	16	8	16	>128
7	0.06	0.004	0.25	2	4	0.03	0.25	>128
8	0.015	0.008	0.12	4	8	16	2	>128
9	0.12	0.008	1	4	4	8	2	>128
10	0.015	0.008	0.25	4	4	8	4	>128

*^a^* ORI, oritavancin; RIF, rifampin; GEN, gentamicin; LZD, linezolid.

**Table 2:** Characteristics of included studies.

Ref.	Study Design	No. of Enrolled Patients	Oritavancin Treatment Protocol	Treatment Outcomes	Follow-Up Duration	Safety Profile of Oritavancin for PJI Cases
[31]	Case report	1	1200 mg intravenous every 7 days ×6 (sole therapy)	Patient mobility/functional status: The patient was ambulating independently without symptoms.	10 months	Not mentioned
[24]	Retrospective cohort, single-center	5	1200 mg intravenous	Specific outcomes for the individual PJI cases were not detailed	- Total duration of patient follow-up: 1 year after the last dose of oritavancin	Three experienced adverse reactions: a 56-year-old female developed itching, hives, and throat swelling during her first infusion, which required cessation of therapy; a 60-year-old female had a rash after her third dose, which appeared post-infusion; and a 46-year-old female experienced shaking, chills, vomiting, and back pain during her third infusion, necessitating immediate discontinuation.
[32]	Case series (single center)	2	1200 mg intravenous, multidose	Both patients successfully treated	Insufficient follow-up information	Not mentioned
[29]	Case report	1	1200 mg of oritavancin, 10 doses until the report was published, the patient is under long-lasting suppressive antimicrobial therapy with 1 dose every 28 days.	No pain with completely recovered joint mobility, complete healing of the fistula and the disappearance of the underlying abscess.	28 weeks	None
[28]	Retrospective cohort, Case series, Multicenter (number of centers not specified)	2	1st case 1200 mg of oritavancin, 8 doses 2nd case 1200 mg/month for 22 months	1st case—recurrence of knee stiffness and persistent VRE infection. 2nd case—sustained symptom-free status	195 days/22 months	None
[30]	Retrospective cohort study; Multicenter study	3	2 patients 1200 mg single dose, 1 patient 1200 mg 2 doses	2 cured patients	Not specifically mentioned	None

## Data Availability

No new data were created or analyzed in this study. Data sharing is not applicable to this article.

## Abbreviations

The following abbreviations are used in this manuscript:

PJIs	periprosthetic joint infections
MRSE	methicillin-resistant *Staphylococcus epidermidis*
MRSA	methicillin-resistant *Staphylococcus aureus*
TDM	therapeutic drug monitoring
